# Comparison of DNA Methylation in Vibrio vulnificus Cells Grown in Human Serum with Those Grown in Seawater

**DOI:** 10.1128/MRA.00419-19

**Published:** 2019-07-18

**Authors:** James W. Conrad, Valerie J. Harwood

**Affiliations:** a Department of Integrative Biology, University of South Florida, Tampa, Florida, USA; Georgia Institute of Technology

## Abstract

The chromosomal methylation statuses of the highly virulent Vibrio vulnificus strain CMCP6 grown in human serum and in seawater are compared here. Growth in seawater resulted in ∼4 times as much methylation as that in human serum, primarily N^4^-methylcytosines.

## ANNOUNCEMENT

The virulence of Vibrio vulnificus is poorly understood, and the only definitive virulence factor is the presence of a capsule (1–3). DNA methylation has been implicated in the virulence of several other bacterial species (4–7). Changes in methylation sites may enable bacteria to respond to changes in their environment by altering gene expression (4, 8, 9). We hypothesized that the transition from an aquatic habitat to infection in a human could require altered DNA methylation, which we explored by growing the bacterium in human serum amended with iron (to mimic hemochromatosis) and in seawater (10).

Here, we have sequenced the methylome of the highly virulent and well-studied V. vulnificus strain CMCP6 (NCBI:txid216895), which was originally isolated from an infected patient in Korea (11). Bacteria were grown to late exponential phase in brain heart infusion broth, pelleted, and resuspended in phosphate-buffered saline at pH 7.5 (repeated once to wash cells). Normal pooled human serum (MP Biomedicals) was amended with ferric citrate to a final concentration of 0.896 mM Fe (12). Seawater (pH 7.8; salinity, 2%) was collected from Hudson Beach, Florida, and sterilized using a hollow fiber ultrafilter (Rexeed 25S). Cultures were established at a starting concentration of 10^7^ CFU/ml in 10 ml of serum or 40 ml of seawater and were shaken for 210 min at 37°C and 30°C, respectively. DNA was extracted with a blood and cell culture DNA minikit (Qiagen).

Preparation of 10-kb libraries and sequencing were performed at the National Center for Genome Resources in Santa Fe, New Mexico. Each sample was sequenced on two single-molecule real-time (SMRT) cells using PacBio RS II P5-C3 chemistry. *De novo* genome assembly proceeded with all four SMRT cells using RS_HGAP_Assembly.3 in SMRT Analysis 2.3.0 with default parameters, resulting in >1.3 million mapped subreads (average subread length, 2,053 bp) and eight contigs (six spurious) (13). Two contigs corresponding to chromosomes 1 and 2 (14) with a total length of 5,199,228 nucleotides (46.75% GC content; 499.3× coverage; quality value [QV], 48.7) were obtained. The assembled genome was 72,430 nucleotides and 72,532 nucleotides longer than the V. vulnificus CMCP6 genomes in the Integrated Microbial Genomes and Microbiomes system and NCBI, respectively.

Methylated bases and motifs were identified in the *de novo* assembly using RS_Modification_and_Motif_Analysis with default parameters (modification QV, 30). All identified methylated motifs had ≥70-fold or 180-fold coverage in human serum and seawater, respectively (Table 1). Seawater treatment resulted in ∼4 times as many methylated bases (primarily N^4^-methylcytosine) as human serum did (447,389 versus 142,369, respectively).

**TABLE 1:** Motifs in human serum and seawater detected using SMRT Analysis v2.3.0

Motif^*a*^	Type	Percent modified in:		No. of motifs in genome	Motif score for:	
		Human serum	Seawater		Human serum	Seawater
G**A**TC	m6A	98.74	98.89	44,386	111.96	239.94
GCC**A**N_9_TCC	m6A	98.32	98.32	537	109.05	242.73
GG**A**N_9_TGGC	m6A	97.39	97.58	537	109.26	239.93
**A**KGYAVYW	m6A	18.80	NA^*b*^	6,283	48.53	NA
**A**KGYASYW	m6A	NA	27.58	3,985	NA	68.71
**A**DDTRGCAD	m6A	18.54	22.16	2,956	47.74	69.04
**C**SNNNNNG	m4C	6.67	NA	264,532	47.59	NA
**C**WGNNVNG	m4C	3.98	NA	50,788	43.90	NA

a Modified bases are bolded as follows: **A**, N^6^-methyladenine (m6A); **C**, N^4^-methylcytosine (m4C). The methylated bases on the complementary strand are underlined.

b NA, not applicable.

### Data availability.

These whole-genome sequencing data have been deposited in the Sequence Read Archive under the project accession number PRJNA503483. The GenBank genome accession numbers are CP037931 and CP037932 for chromosomes 1 and 2, respectively. The accession numbers for the raw sequencing reads of V. vulnificus CMCP6 are SAMN10360788 and SAMN10360789 for human serum and seawater, respectively.
